# Diagnostic omission errors in acute paediatric practice: impact of a reminder system on decision-making

**DOI:** 10.1186/1472-6947-6-37

**Published:** 2006-11-06

**Authors:** Padmanabhan Ramnarayan, Andrew Winrow, Michael Coren, Vasanta Nanduri, Roger Buchdahl, Benjamin Jacobs, Helen Fisher, Paul M Taylor, Jeremy C Wyatt, Joseph Britto

**Affiliations:** 1 Children's Acute Transport Service (CATS), 44B Bedford Row, London, WC1H 4LL, UK; 2 Department of Paediatrics, Kingston General Hospital, Galsworthy Road, Kingston-upon-Thames, KT2 7QB, UK; 3 Department of Paediatrics, St Mary's Hospital, Paddington, London, W2 1NY, UK; 4 Department of Paediatrics, Watford General Hospital, Vicarage Road, Watford, WD18 0HB, UK; 5 Department of Paediatrics, Hillingdon Hospital, Pield Heath Road, Middlesex, UB8 3NN, UK; 6 Department of Paediatrics, Northwick Park Hospital, Watford Road, Harrow, Middlesex, HA1 3UJ, UK; 7 Isabel Healthcare Ltd, Po Box 244, Haslemere, Surrey, GU27 1WU, UK; 8 Centre for Health Informatics and Multiprofessional Education (CHIME), Archway Campus, Highgate Hill, London, N19 5LW, UK; 9 Health Informatics Centre, The Mackenzie Building, University of Dundee, Dundee, DD2 4BF, UK

## Abstract

**Background:**

Diagnostic error is a significant problem in specialities characterised by diagnostic uncertainty such as primary care, emergency medicine and paediatrics. Despite wide-spread availability, computerised aids have not been shown to significantly improve diagnostic decision-making in a real world environment, mainly due to the need for prolonged system consultation. In this study performed in the clinical environment, we used a Web-based diagnostic reminder system that provided rapid advice with free text data entry to examine its impact on clinicians' decisions in an acute paediatric setting during assessments characterised by diagnostic uncertainty.

**Methods:**

Junior doctors working over a 5-month period at four paediatric ambulatory units consulted the Web-based diagnostic aid when they felt the need for diagnostic assistance. Subjects recorded their clinical decisions for patients (differential diagnosis, test-ordering and treatment) before and after system consultation. An expert panel of four paediatric consultants independently suggested clinically significant decisions indicating an appropriate and 'safe' assessment. The primary outcome measure was change in the proportion of 'unsafe' workups by subjects during patient assessment. A more sensitive evaluation of impact was performed using specific validated quality scores. Adverse effects of consultation on decision-making, as well as the additional time spent on system use were examined.

**Results:**

Subjects attempted to access the diagnostic aid on 595 occasions during the study period (8.6% of all medical assessments); subjects examined diagnostic advice only in 177 episodes (30%). Senior House Officers at hospitals with greater number of available computer workstations in the clinical area were most likely to consult the system, especially out of working hours. Diagnostic workups construed as 'unsafe' occurred in 47/104 cases (45.2%); this reduced to 32.7% following system consultation (McNemar test, p < 0.001). Subjects' mean 'unsafe' workups per case decreased from 0.49 to 0.32 (p < 0.001). System advice prompted the clinician to consider the 'correct' diagnosis (established at discharge) during initial assessment in 3/104 patients. Median usage time was 1 min 38 sec (IQR 50 sec – 3 min 21 sec). Despite a modest increase in the number of diagnostic possibilities entertained by the clinician, no adverse effects were demonstrable on patient management following system use. Numerous technical barriers prevented subjects from accessing the diagnostic aid in the majority of eligible patients in whom they sought diagnostic assistance.

**Conclusion:**

We have shown that junior doctors used a Web-based diagnostic reminder system during acute paediatric assessments to significantly improve the quality of their diagnostic workup and reduce diagnostic omission errors. These benefits were achieved without any adverse effects on patient management following a quick consultation.

## Background

Studies suggest that a significant proportion of adverse events in primary as well as in secondary care result from errors in medical diagnosis [1-3]; diagnostic errors also constitute the second leading cause for malpractice suits against hospitals [4]. Specialities such as primary care and emergency medicine have specifically been identified as high risk areas for diagnostic mishaps, where cognitive biases in decision making contribute to errors of omission, resulting in incomplete workup and 'missed diagnoses' [5-7]. Adverse events are also commoner in extremes of age, such as paediatric patients and the elderly [8,9]. Diagnostic decision support systems (DDSS), computerised tools that provide accurate and useful patient- and situation-specific advice have been proposed as a technological solution for the reduction of diagnostic errors in practice [10]. Although a number of 'expert diagnostic systems' exist currently, a recent systematic review showed that these systems were less effective in practice than systems that provided preventive care reminders and prescription advice [11]. To a large extent, this may be because most latter systems were integrated into an existing electronic medical record (EMR), enabling effortless and frequent use by clinicians; in contrast, expert DDSS such as Quick Medical Reference (QMR), ILIAD and MEDITEL-PEDS were typically used in stand-alone fashion [12-14]. Due to a lengthy data input process, considerable clinician motivation and effort was required for their regular use, leading to infrequent usage [15]. As a result, busy clinical areas have been poorly served by existing DDSS.

Attempts to integrate diagnostic decision support into an EMR have been sporadic [16,17], mainly limited by the difficulties associated with converting a complex clinical narrative into structured clinical data in a standard EMR, especially for specialities such as paediatrics and emergency medicine. It appears likely that in the medium term, effortless provision of decision support for busy clinical areas at high-risk for diagnostic error seems possible only through alternative approaches. A Web-based paediatric DDSS that permits rapid use in a busy clinical environment by using natural language free text data entry has been recently described [18,19]. Its underlying knowledge base consists of textual descriptions of diseases; using statistical natural language processing, the DDSS matches clinical features to disease descriptions in the database. This approach is similar to that adopted by the RECONSIDER program [20]. Diagnostic suggestions are displayed in sets of 10 up to a maximum of 30, and arranged by body system (e.g. cardiology) rather than by clinical probability. Between 2001 and 2003, >15,000 users registered for its use, 10% of whom used it on >10 separate occasions, resulting in >60,000 distinct user log-ins (personal communication). Thus, although poor usage has been a major confounding factor during evaluations of the clinical benefits of a number of DDSS [21], Isabel usage statistics led us to believe that a study evaluating its clinical impact would permit the assessment of its benefits and risks to be interpreted with confidence, and provide useful insights into the user-DDSS dynamic. Results from an independent email questionnaire survey also suggested that most regular users in the UK found it helpful during patient management [22].

In this study, we aimed to measure the clinical impact of the Isabel system on diagnostic decision making. We hypothesised that lessons learnt from our evaluation study could be generalised to the design, implementation and evaluation of other stand-alone DDSS, and clarify the risks associated with the use of such a system in real life. Diagnostic suggestions were provided to junior doctors during acute paediatric assessments in which they experienced diagnostic uncertainty.

## Methods

The study was co-ordinated from St Mary's Hospital, Imperial College London, and was approved by the London multi-centre research ethics committee (MREC/02/2/70) and relevant local research ethics committees.

### Study centres

From a list of non-London district general hospitals (DGH) at which >4 individual users were registered in the Isabel database, four paediatric departments (two university-affiliated DGHs and two DGHs without official academic links) were enrolled, based on logistical and clinical reasons – all sites were <100 miles driving distance from London; three were geographically clustered in the Eastern Region and two sites were separated by large distances from regional tertiary centres. The baseline characteristics of each of the participating centres are detailed in table 1. None of the study centres were involved in the development of the DDSS.

**Table 1:** Characteristics of participating paediatric departments

	**Centre A**	**Centre B**	**Centre C**	**Centre D**
Nature	university	dgh	dgh	university
24 hours dedicated PAU	no*	yes	yes	yes
Annual PAU attendance	4194	4560	4780	4800
Number of junior doctors	31	21	12	16
Number of consultants (acute)	10 (6)	7 (4)	7 (4)	11 (7)
Computers in PAU	2	1	1	3
Metropolitan area	yes	mixed	no	no
Dist to tertiary centre (miles)	<25	26	71	69
Clinical activity (PAU attendances per hour PAU open)	1.1	0.53	0.54	0.67
Computer accessibility index (available computers per unit clinical activity)	1.8	1.9	1.85	4.5
Senior support (number of acute consultants per subject enrolled)	0.2	0.2	0.3	0.4

Abbreviations: dgh = district general hospital, PAU = paediatric assessment unit

* PAU open from 0800 to 0000 only

### Study participants

All junior doctors (Senior House Officers [interns] and Registrars [residents]) in substantive posts at each of the participating paediatric departments between December 2002 and April 2003 were enrolled after informed verbal consent. Consultants (attending physicians) and locum doctors were excluded.

### Study patients

All children (age 0–16 years) presenting with an acute medical complaint, and assessed by a junior doctor in a designated Paediatric Assessment Area/Ambulatory Unit (PAU), were eligible for DDSS use. Outpatients, re-attendances for ward follow up, and day cases were ineligible. Based on subjects' feedback collected prior to the study start date, we made a pragmatic decision to allow junior doctors to selectively consult the DDSS only for patients in whom they experienced diagnostic uncertainty. This latter subset formed the actual study population.

### Study design and power

Our study was a within-subject 'before and after' evaluation in which each study subject acted as their own control. Participants explicitly recorded their diagnostic workup and clinical plans (tests and treatment) for cases before seeking DDSS advice. Following real-time use, subjects either decided to act on system advice (by recording their revised diagnostic workup and clinical plans) or chose not to, thus ending the consultation. On the basis of a pilot study in an experimental setting, we calculated that the trial required data from 180 cases to detect a 33% reduction in clinically 'unsafe' diagnostic workups (80% power; type I error 5%). We defined diagnostic workups as being 'unsafe' if they deviated from a 'minimum gold standard' provided by an independent expert panel.

### Intervention

#### Decision support system

**Figure 1:**
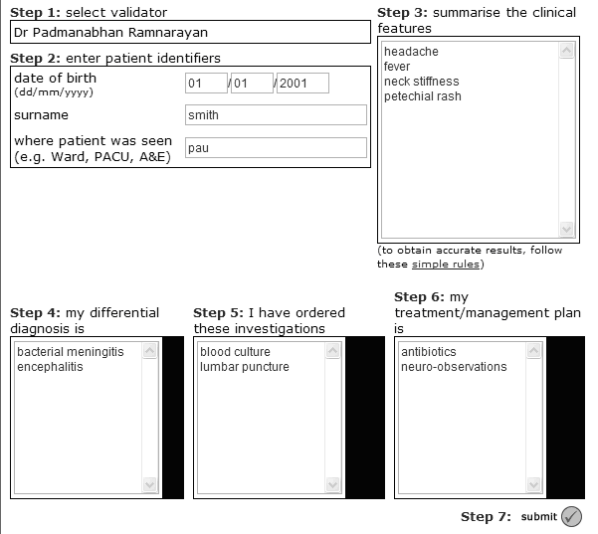
This figure shows screen 1 during the DDSS usage during the study. This page is loaded following a successful log-in, which is initiated by clicking a designated icon on each study computer at each participating centre. Information collected automatically at this stage includes date and time of screen 1 display; patient identifiers; subject identifiers; clinical features of patient, and initial diagnostic workup and tests as well as treatments.

**Figure 2:**
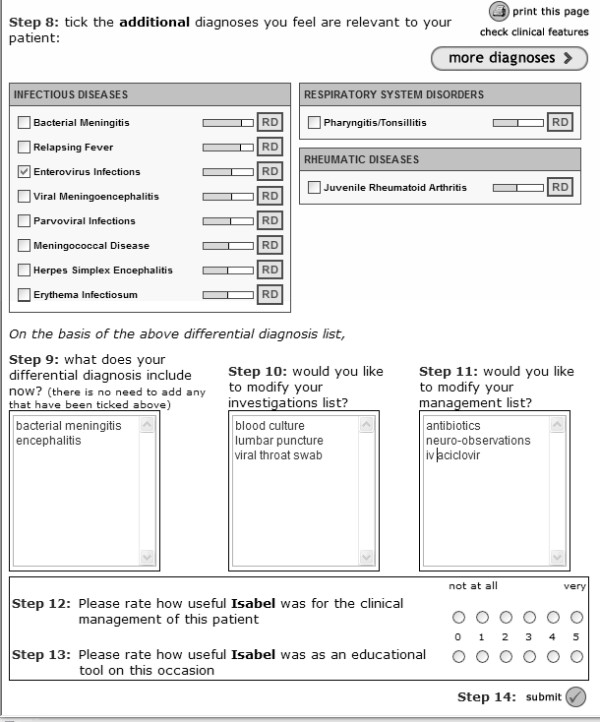
This figure shows screen 2, which is displayed following submission of the information entered on screen 1. The subject has the opportunity to revise their decisions, including diagnoses and tests and treatments. A brief survey attempts to capture the user's satisfaction with respect to educational value as well as clinical utility. Information submitted following this page completes the episode of data collection.

A limited, secure (128-bit SSL encryption) and password-protected trial version was used. This differed from the publicly accessible DDSS – only the first 10 diagnostic suggestions were displayed, and reading material related to each diagnosis was not made available. The trial version was accessible only via a designated shortcut icon placed on each computer present at study commencement within the participating PAUs. This mechanism utilised cookies, and automatically facilitated identification of the centre at which the request originated, allowing subjects to access the system without an additional log-in procedure. On the trial DDSS, data were captured in two consecutive screens (figures 1 and 2). On screen 1, subjects recorded the patient's clinical details in their own words, and their own diagnostic workup and clinical plans (pre-DDSS). Based on the clinical details submitted in screen 1, diagnostic suggestions were displayed on screen 2. Subjects had the choice to revise their original diagnostic workup and clinical plans by adding or deleting decisions at this stage. It was not possible to go back from screen 2 to screen 1. The consultation ended when clinical decisions from screen 2 (unmodified or revised) were finally submitted, or after >2 hours of inactivity.

#### Training

Three separate group training sessions were organised by one investigator (PR) at each centre one month before the study start date, coinciding with weekly mandatory departmental teaching sessions. At each session, subjects used the trial DDSS with practice cases created for the study. Sessions were repeated twice during the study period to recruit and train new post-holders.

### Outcome measures

The primary outcome measure was change in the proportion of 'unsafe' diagnostic workups following DDSS consultation. We defined 'unsafe' workups as instances in which subjects' diagnostic workup (pre- and post-DDSS consultation) deviated from a 'minimum gold standard' provided by an independent expert panel of clinicians.

Inclusion of the correct discharge diagnosis in the diagnostic workup (pre- and post-Isabel consultation), quality scores for diagnostic workup and clinical action plans; time taken by subjects to complete system usage; number of diagnoses included in the diagnostic assessment pre- and post-DDSS; inappropriate tests and treatments ordered by subjects following system advice and significant decisions deleted following consultation were examined as secondary outcome measures.

### Data collection

Clinical data were collected during the study period (December 2002-April 2003) by two complementary methods – automatically from trial DDSS logs, and manually by a single research assistant (HF). Data collected by the trial website during system use is shown in table 2. At the end of each clinical consultation, the user indicated how useful the diagnostic advice provided had been for a) patient management and b) as an educational exercise. This was recorded on a Likert-scale from 0–5 (not useful to extremely useful). All subjects were sent two rounds of email and postal questionnaires each to collect feedback at trial completion.

**Table 2:** Study data automatically collected by the DDSS logs

**Patient details**
Surname
Date of birth
Age group (neonate, infant, child or adolescent)
Sex
**User details**
Centre code (based on identity of icon clicked)
Subject identity (including an option for anonymous)
Subject grade
**Operational details**
Date and time of usage (log in, submission of each page of data)
Unique study ID assigned at log in
**Clinical details**
Patient clinical features at assessment
Doctor's differential diagnosis (pre-ISABEL)
Doctor's investigation plan (pre-ISABEL)
Doctor's management plan (pre-ISABEL)
Isabel list of differential diagnoses
Diagnoses selected from Isabel list by user as being relevant
Doctor's differential diagnosis (post-ISABEL)
Doctor's investigation plan (post-ISABEL)
Doctor's management plan (post-ISABEL)
**Survey details**
Satisfaction score for patient management
Satisfaction score for educational use

The research assistant obtained study patients' medical records that matched the patient identifiers collected automatically from the trial website during system use. It was not possible to use partial or invalid entries to match medical records. Copies of available medical records were made such that information was available only up to the point of DDSS use. Copies were anonymised by masking patient and centre details. Diagnostic workup and clinical plans recorded by subjects on the trial website were verified for each case against entries in the medical records and in hospital laboratory systems. Discharge diagnoses were collected from routinely collected coding data for all study patients, and additionally from discharge summaries where available. Discharge diagnoses were validated by a consultant involved in study conduct at each centre. In addition, limited demographic and clinical details of all eligible patients at each study centre were collected from hospital administrative data.

A panel of four consultant paediatricians independently examined study medical records, in which subjects' clinical decisions were masked to ensure blinding. In the first instance, each panel member provided a list of 'clinically significant' diagnoses, tests and treatments (the latter two were collectively termed 'clinical action plans') for each case that would ensure a safe clinical assessment. The absence of 'clinically significant' items in a subject's workup was explicitly defined during panel review to represent inappropriate clinical care. For this reason, the panel members did not include all plausible diagnoses for each case as part of their assessment, and instead focused on the minimum gold standard. Using this list as a template, the appropriateness of each decision suggested by subjects for each case was subsequently scored by the panel in blinded fashion using a previously validated scoring system [23]. This score rewarded decision plans for being comprehensive (sensitive) as well as focussed (specific). 25% of medical records were randomly assigned to all four panel members for review; a further 20% was assigned to one of the six possible pairs (i.e. slightly more than half the records were assessed by a single panel member). Clinically significant decisions (diagnoses, tests and treatments) were collated as a 'minimum gold standard' set for each case. For cases assessed by multiple panel members, significant decisions provided by a majority of assessors were used to form the gold standard set. Concordance between panel members for clinical decisions was moderate to good, as assessed by the intra-class correlation co-efficient for decisions examined by all four members (0.70 for diagnoses, 0.47 for tests and 0.57 for treatments).

### Analysis

We analysed study data from two main perspectives: operational and clinical. For operational purposes, we defined each attempt by a subject to log into the DDSS by clicking on the icon as a 'DDSS attempt'. Each successful display of screen 1 was defined as a 'successful log in'; a unique study identifier was automatically generated by the trial website for each successful log in. Following log in, DDSS usage data was either 'complete' (data were available from screens 1 and 2) or 'incomplete' (data were available from screen 1 only, although screen 2 may have been displayed to the subject). Time spent by the user processing system advice was calculated as the difference between the time screen 2 was displayed and the end of the consultation (or session time out).

In the first instance, we used McNemar's test for paired proportions to analyse the change in proportion of 'unsafe' diagnostic workups. In order to account for the clustering effects resulting from the same subject assessing a number of cases, we also calculated a mean number of 'unsafe' diagnostic workups per case attempted for each subject. Change in this variable following DDSS consultation was analysed using two-way mixed-model analysis of variance (subject grade being between-subjects factor and occasion being within-subjects factor). In order to exclude re-thinking effect as an explanation for change in the primary outcome variable, all episodes in which there was a difference between the workup pre- and post-DDSS consultation were examined. If diagnoses that were changed by subjects were present in the Isabel suggestion list, it could be inferred that the DDSS was responsible for the change. A more objective marker of clinical benefit was assessed by examining whether the post-Isabel diagnostic workup (but not the pre-Isabel workup) included the discharge diagnosis. We analysed changes in pre- and post-DDSS diagnostic quality scores, as well as clinical action plan scores, using subjects as the unit of analysis. We tested for statistical significance using one way analysis of variance (grade was the between-subjects factor) to provide a sensitive measure of changes in diagnostic workup, and tests and treatments. The median test was used to examine differences between grades in system usage time. Diversity of suggestions displayed by the DDSS during the study, and therefore its dynamic nature, was assessed by calculating the number of unique diagnoses suggested by the system across all episodes of completed usage (i.e. if the diagnostic suggestions remained constant irrespective of case characteristics, this number would be 10). Statistical significance was set for all tests at p value <0.05.

Subjects were expected to use the DDSS in only a subset of eligible patients. In order to fully understand the characteristics of patients in whom junior doctors experienced diagnostic difficulty and consulted the DDSS, we examined this group in more detail. We analysed available data on patient factors (age, discharge diagnosis, outcome of assessment and length of inpatient stay if admitted), user factors (grade of subject), and other details. These included the time of system usage (daytime: 0800–1800; out-of-hours: 1800-0800), centre of use and its nature (DGH vs university-affiliated hospital), an index of PAU activity (number of acute assessments per 60 min period the PAU was functional), computer accessibility index (number of available computers per unit PAU activity) and level of senior support (number of acute consultants per subject). We subsequently aimed to identify factors that favoured completion of DDSS usage using a multiple logistic regression analysis. Significant variables were identified by univariate analysis and entered in forward step-wise fashion into the regression model. Characteristics of patients where subjects derived clinical benefit with DDSS usage were also analysed in similar fashion. We correlated subjects' own perception of system benefit (Likert style response from user survey) with actual benefit (improvement in diagnostic quality score) using the Pearson test. Qualitative analysis of feedback from subjects provided at the end of the study period was performed to provide insights into system design and user interface.

## Results

**Figure 3:**
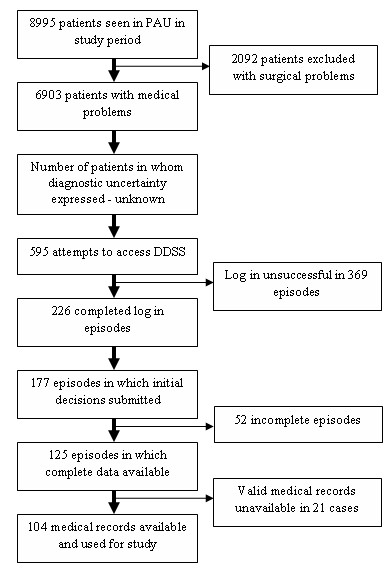
Flow diagram of patients and data through the study.

**Figure 4:**
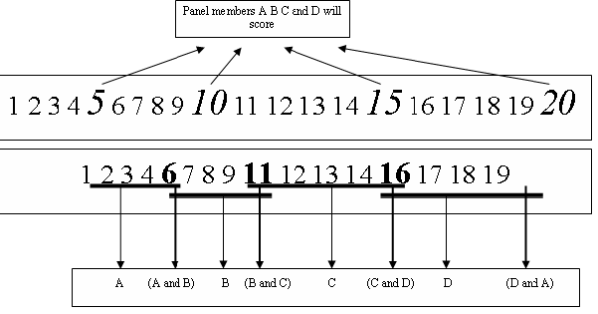
Allocation of medical records for expert panel assessment consisting of four raters. All panel members rated 25% of cases and two raters (six possible pairs) rated an additional 20% of cases.

During the 5-month study period, 8995 children were assessed in the 4 PAUs; 76.7% (6903/8995 children) presented with medical problems and were eligible for inclusion in the study. Subjects attempted to seek diagnostic advice on 595 separate occasions across all centres (8.6%). Subjects successfully logged in only in 226 episodes. Data were available for analysis in 177 cases (complete data in 125 cases; screen 1 data only in an additional 52 cases). The summary of flow of patients and data through the study is illustrated in figure 3. Centre-wise distribution of attrition in DDSS usage is demonstrated in table 3. Medical records were available for 104 patients, and were examined by the panel according the allocation scheme shown in figure 4.

**Table 3:** Centre-wise attrition of DDSS usage and study data

	**Centre A**	**Centre B**	**Centre C**	**Centre D**	**Total**
Patients seen in PAU	2679	1905	1974	2437	**8995**
Medical patients seen in PAU	2201	1383	1405	1914	**6903**
Number eligible for diagnostic decision support	unknown	unknown	unknown	unknown	unknown
DDSS attempts	338	118	52	87	**595**
DDSS successful log in	unknown	unknown	unknown	unknown	226*
Step 1 completed†	47	26	45	59	**177**
Steps 1&2 completed	30	25	20	50	**125**
Medical records available	24	24	16	40	**104**

* Each successful log in was automatically provided a unique study identifier which was not centre-specific. The number of successful log-ins was thus calculated as the total number of study identifiers issued by the trial website.

† Step 1 completed indicates that following successful log-in, the subject entered data on the first screen, i.e. patient details.

The largest subset of patients in whom the DDSS was consulted belonged to the 1–6 year age group (61/177, 34.5%). The mean age of patients was 5.1 years (median 3.3 years). The DDSS was most frequently used by SHOs (126/177, 71.2%). Although 25% of all eligible patients were seen on PAUs outside the hours of 08:00 to 18:00, more than a third of episodes of system usage fell outside the hours of 0800–1800 (64/177, 36.2%). Subjects at Centre D used the DDSS most frequently (59/177, 33.3%). In general, usage was greater at university-affiliated hospitals than at DGHs (106/177, 59.9%). Discharge diagnosis was available in 77 patients. The commonest diagnosis was non-specific viral infection; however, a wide spread of diagnoses was seen in the patient population. 58 patients on whom the DDSS was consulted were admitted to hospital wards, the rest were discharged home following initial assessment. Inpatients stayed in hospital for an average of 3.7 days (range: 0.7–26 days). Clinical characteristics of patients on whom Isabel was consulted are summarised in table 4. A list of discharge diagnoses in table 5 indicates the diverse case mix represented in the study population.

**Table 4:** Characteristics of patients in whom Isabel was consulted

Factor	Number of DDSS consultation episodes (completed episodes)
PATIENT FACTORS	
**Age (n = 177)**	
Neonate	19
Infant	33
Young child (1–6 yrs)	61
Older child (6–12 yrs)	38
Adolescent	26
	
**Primary diagnostic group (n = 77)**	
Respiratory	9
Cardiac	0
Neurological	6
Surgical	3
Rheumatology	5
Infections	31
Haematology	3
Other	20
	
**Outcome (n = 104)**	
IP admission	58
Discharge	46
	
USER FACTORS	
**Grade (n = 177)**	
SHO	126 (79)
Registrar	51 (46)
	
OPERATIONAL FACTORS **(n = 177)**	
**Time of use**	
In hours (0800–1800)	113 (84)
Out of hours (1800-0800)	64 (41)
	
**Centre**	
A	47 (30)
B	26 (25)
C	45 (20)
D	59 (50)

**Table 5:** Discharge diagnoses in children in whom the diagnostic aid was consulted

Diagnosis	Number of patients
Viral infection	8
Acute lymphadenitis	3
Viral gastroenteritis	3
Pelvic region and thigh infection	3
Epilepsy	3
Acute lower respiratory infection	3
Allergic purpura	2
Acute inflammation of orbit	2
Chickenpox with cellulitis	2
Gastroenteritis	2
Rotaviral enteritis	2
Feeding problem of newborn	2
Syncope and collapse	2
Lobar pneumonia	2
Kawasaki disease	2
Abdominal pain	2
Angioneurotic oedema	1
Erythema multiforme	1
Constipation	1
Irritable bladder and bowel syndrome	1
Coagulation defect	1
G6PD deficiency	1
Sickle cell dactylitis	1
Cellulitis	1
Clavicle osteomyelitis	1
Kerion	1
Labyrinthitis	1
Meningitis	1
Myositis	1
Purpura	1
Scarlet fever	1
Staphylococcal scalded skin syndrome	1
Mitochondrial complex 1 deficiency	1
Adverse drug effect	1
Eye disorder	1
Musculoskeletal back pain	1
Trauma to eye	1
Disorders of bilirubin metabolism	1
Foetal alcohol syndrome	1
Neonatal erythema toxicum	1
Physiological jaundice	1
Stroke	1
Acute bronchiolitis	1
Acute upper respiratory infection	1
Asthma	1
Hyperventilation	1
Juvenile arthritis with systemic onset	1
Polyarthritis	1
Reactive arthropathy	1
Anorectal anomaly	1

**Figure 5:**
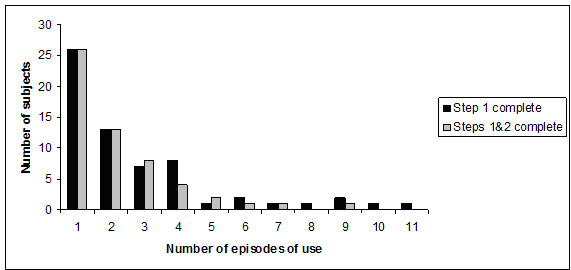
DDSS usage data shown as distribution of number of subjects by episodes of system use.

80 subjects enrolled during the study. 63/80 used the system to record some patient data (mean 2, range: 1–12); 56/80 provided complete patient information and their revised decisions post-DDSS consultation (mean 2, range: 1–6). Due to limitations in the trial website design, it was unclear how many of the subjects who did not use the system during the study period (17/80) had attempted to access the DDSS and failed. It was evident that a small number of subjects used the DDSS on multiple (>9) occasions but did not provide their revised clinical decisions, leading to incomplete system use. System usage data with respect to subjects is shown in figure 5.

### 'Unsafe' diagnostic workups

104 cases in which medical records were available were analysed. Before DDSS consultation, 'unsafe' diagnostic workups occurred in 47/104 cases (45.2%); they constituted episodes in which all clinically significant diagnoses were not considered by subjects during initial decision making. Overall, the proportion of 'unsafe' workups reduced to 32.7% (34/104 cases) following DDSS consultation, an absolute reduction of 12.5% (McNemar test p value <0.001, table 6). In a further 5 cases, appropriate diagnoses that were missing in subjects' workup did form part of Isabel suggestions, but were ignored by subjects during their review of DDSS advice. In 11/13 cases in which 'unsafe' diagnostic workups were eliminated, the DDSS was used by SHOs. Mean number of 'unsafe' workups per case reduced from 0.49 to 0.32 post-DDSS consultation among subjects (p < 0.001); a significant interaction was demonstrated with grade (p < 0.01). Examination of the Isabel suggestion list for cases in which there was a difference between pre- and post-DDSS workup showed that in all cases, the additional diagnoses recorded by subjects formed part of the system's advice. Similar results were obtained for clinical plans, but smaller reductions were observed. In 3/104 cases, the final diagnosis for the patient was present in the post-DDSS list but not in the pre-DDSS list, indicating that diagnostic errors of omission were averted following Isabel advice.

**Table 6:** Reduction in unsafe diagnostic workups following DDSS consultation (n = 104)

	Pre-DDSS consultation	Post-DDSS consultation	Relative Reduction (%)
Unsafe diagnostic workup			
SHO	28	17	39.3
Registrar	19	17	10.5

### Diagnostic quality scores

104 cases assessed by 51 subjects in whom medical records were available were analysed. Mean diagnostic quality score across all subjects increased by 6.86 (95% CI 4.0–9.7) after DDSS consultation. The analysis of variance model indicated that there was no significant effect of grade on this improvement (p 0.15). Similar changes in clinical plan scores were smaller in magnitude (table 7).

**Table 7:** Changes in mean quality scores for diagnostic workup and clinical action plans

	SHO	Registrar	Overall
Diagnostic quality score change (SD)	8.3 (11.6)	3.8 (6.1)	6.9 (10.3)
Clinical action plan score change (SD)	1.4 (6.3)	1.7 (7.4)	1.5 (6.7)

### Usage time

Reliable time data were available in 122 episodes. Median time spent on system advice was 1 min 38 sec (IQR 50 sec – 3 min 21 sec). There was no significant difference between grades with respect to time spent on screen 2 (median test, p = 0.9). This included the time taken to process DDSS diagnostic suggestions, record changes to original diagnostic workup and clinical plans, and to complete the user satisfaction survey.

### Impact on clinical decision making

Pre-DDSS, a mean of 2.2 diagnoses were included in subjects' workup; this rose to 3.2 post-DDSS. Similarly, the number of tests ordered also rose from 2.7 to 2.9; there was no change in the number of treatment steps. Despite these increases, no deleterious tests or treatment steps were added by subjects to their plans following DDSS consultation. In addition, no clinically significant diagnoses were deleted from their original workup after Isabel advice.

Using forward step-wise regression analysis, grade of subject (registrar), time of system usage (in-hours), centre identity, senior support and computer accessibility index were identified as independent factors predicting completion of DDSS usage. Patients in whom actual benefit was demonstrated on diagnostic decision making were more likely to stay longer in hospital.

469 unique diagnostic suggestions were generated by the DDSS during its use on 125 cases. This represented a high degree of diversity of responses appropriate for the diverse case mix seen in this study – a static list would have consisted of the same 10 diagnoses, and a unique set of suggestions for each single episode of use would have generated 1250 distinct suggestions.

### User perception

**Figure 6:**
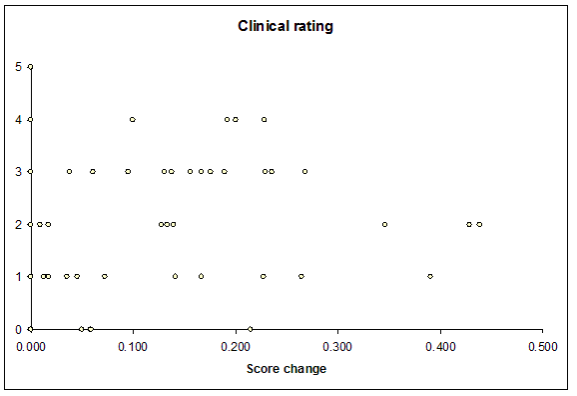
Correlation between user perception of system utility and change in diagnostic quality score.

Data were available in 125 cases in which subjects completed DDSS usage. Mean satisfaction score for patient management was 1.6 (95% CI 1.4–1.96); for Isabel use as an educational adjunct, this was higher (2.4, 95% CI 1.98–2.82). There was moderate correlation between subjects' perception of DDSS usefulness in patient management and actual increment in diagnostic quality score (r value 0.28, p value 0.0038, figure 6). Feedback from questionnaires indicated that many subjects found the trial website cumbersome to use in real time since it forced them to record all their decisions prior to advice, thus taking up time during patient assessment. This was especially problematic since many subjects had used Isabel in its original form. A number of subjects were dissatisfied with computer access during the trial; these related to unavailability of passwords to access the Internet, slow computer connections, unavailability of adequate workstations at the point of clinical use and lack of infrastructure support. Another theme that emerged from user feedback involved the lack of access to reading material on diagnoses during the trial period – most users felt this was an important part of the system and the advice provided.

## Discussion

This study demonstrates that diagnostic uncertainty occurs frequently in clinical practice, and that it is feasible for a DDSS, unintegrated into an EMR, to improve the process of diagnostic assessment when used by clinicians in real life practice. We have also shown that this improvement prevented a small but significant number of diagnostic errors of omission. A number of barriers to computer and Internet access in the clinical setting prevented system use in a significant proportion of eligible patients in whom subjects sought diagnostic assistance.

The DDSS studied provided advice in the field of diagnosis, an area in which computerised systems have rarely been shown to be effective. In an early clinical study, Wexler et al showed that consultation of MEDITEL-PEDS, a DDSS for paediatric practice, resulted in a decrease in the number of incorrect diagnoses made by residents [24]. However, subjects did not interact with the DDSS themselves; advice generated by the system was provided to clinicians, and diagnostic decisions were amended by subjects on the basis of the information provided. The impact of QMR was studied in similar consultative fashion: a beneficial impact was demonstrated on diagnostic decisions as well as test ordering [25]. In a subsequent laboratory study examining the impact of two different systems (QMR and ILIAD) on simulated cases, a correct diagnosis was added by subjects to their diagnostic workup in 6.5% episodes [26]. Diagnostically challenging cases were deliberately used; it was not clear that junior clinicians would seek diagnostic advice on similar cases in routine practice. Since the user-DDSS dynamic plays a key role in whether these systems are used and the extent of benefit derived from them [27,28] the above-mentioned studies provide limited information on how clinicians would interact with computerised DDSS to derive clinical benefits in practice, especially in a busy environment.

Our study was notable for utilising a naturalistic design, in which subjects used the Isabel system without extensive training or monitoring, allowing results to be generalised to the clinical setting. This design allowed us to explore the complex interplay between user-DDSS interaction, user decisions in the face of diagnostic advice, and barriers to usage. The DDSS selected was already being used frequently in practice; a number of previous system evaluations have been confounded by inadequate usage. The clinical performance of the DDSS studied has also been previously validated [29]. A preliminary assessment of Isabel impact on subjects' diagnostic decisions has already been made in a simulated environment, results of which closely mirror our current findings [30]. Although the nature and frequency of clinicians' information needs have been previously described, we were able to estimate the need for diagnostic decision support, and characterise the subgroup of patients in whom junior clinicians sought diagnostic advice. Since diagnostic uncertainty only occurs in a subset of acutely ill patients, similar interventions in the future will need to be targeted, rather than being universally applied. However, this has to be balanced against our finding that there was poor correlation between subjects' own perception of system utility and actual clinical benefit, which suggests that a universal approach to usage may be more beneficial. This phenomenon has been previously described [31]. We have also identified that junior doctors, such as SHOs, are more likely to use and benefit from DDSS, including in an educational role. Cognitive biases, of which 'premature closure' and faulty context generation are key examples, contribute significantly to diagnostic errors of omission [32], and it is likely that in combination with cognitive forcing strategies adopted during decision making, DDSS may act as 'safety nets' for junior clinicians in practice [33].

Fundamental deviation in function and interface design from other expert systems may have contributed to the observed DDSS impact on decision-making in this study. The provision of reminders has proved highly effective in improving the process of care in other settings [34]. Rapid access to relevant and valid advice is crucial in ensuring usability in busy settings prone to errors of omission – average DDSS consultation time during this study was <2 minutes. It also appears that system adoption is possible during clinical assessment in real time with current computer infrastructure, providing an opportunity for reduction in diagnostic error. EMR integration would allow further control on the quality of the clinical input data as well as provision of active decision support with minimum extra effort; such an interface has currently been developed for Isabel and tested with four commercial EMRs [35]. Such integration facilitates iterative use of the system during the evolution of a patient's condition, leading to increasingly specific diagnostic advice. A number of other observations are worthy of note: despite an increase in the number of diagnoses considered, no inappropriate tests were triggered by the advice provided; the quality of data input differed widely between users; the system dynamically generated a diverse set of suggestions based on case characteristics; the interpretation of DDSS advice itself was user-dependent, leading to variable individual benefit; and finally, on some occasions even useful advice was rejected by users. User variability in data input cannot be solely attributed to the natural language data entry process; considerable user variation in data entry has been demonstrated even in DDSS that employ controlled vocabularies for input [36]. Further potential benefit from system usage was compromised in this study due to many reasons: unavailability of computers, poor Internet access, and slow network connections frequently prevented subjects from accessing the DDSS. Paradoxically, the need to enter detailed information including subjects' own clinical decisions into the trial website (not required during real life usage) may itself have compromised system usage during the study, limiting the extent to which usage data from the study can be extrapolated to real life.

This study had a number of limitations. Our study was compromised by the lack of detailed qualitative data to fully explore issues related to why users sometimes ignored DDSS advice, or specific cases in which users found the DDSS useful. The comparison of system versus a panel gold standard had its own drawbacks – Isabel was provided variable amount of patient detail depending on the subject who used it, while the panel were provided detailed clinical information from medical notes. Changes in decision making were also assessed at one fixed point during the clinical assessment, preventing us from examining the impact of iterative use of the DDSS with evolving and sometimes rapidly changing clinical information. Due to the before-after design, it could also be argued that any improvement observed resulted purely from subjects rethinking the case; since all appropriate diagnoses included after system consultation were present in the DDSS advice, this seems unlikely. Subjects also spent negligible time between their initial assessment of cases and processing the system's diagnostic suggestions. Our choice of primary outcome focused on improvements in process, although we were also able to demonstrate a small but significant prevention of diagnostic error based on the discharge diagnosis. The link between improvements in diagnostic process and patient outcome may be difficult to illustrate, although model developed by Schiff et al suggests that avoiding process errors will lead to actual errors in some instances, as we have demonstrated in this study [37]. However, in our study design, it was not possible to test whether an 'unsafe' diagnostic workup would directly lead to patient harm. Finally, due to barriers associated with computer access and usage, we were not able to reach the target number of cases on whom complete medical data were available.

## Conclusion

This clinical study demonstrates that it is possible for a stand-alone diagnostic system based on the reminder model to be used in routine practice to improve the process of diagnostic decision making among junior clinicians. Elimination of barriers to computer access is essential to fulfil the significant need for diagnostic assistance demonstrated in this study.

## Competing interests

This study was conducted when the Isabel system was available free to users, funded by the Isabel Medical Charity. Dr Ramnarayan performed this research as part of his MD thesis at Imperial College London. Dr Britto was a Trustee of the medical charity in a non-remunerative post. Ms Fisher was employed by the Isabel Medical Charity as a Research Nurse for this study.

Since June 2004, Isabel has been managed by a commercial subsidiary of the Medical Charity called Isabel Healthcare. The system is now available only to subscribed users. Dr Ramnarayan now advises Isabel Healthcare on research activities on a part-time basis; Dr Britto is now Clinical Director of Isabel Healthcare. Both hold restricted shares in Isabel Healthcare. All other authors declare that they have no competing interests.

## Authors' contributions

PR conceived the study, contributed to the study design, analyzed the data and drafted the manuscript.

BJ assisted with data collection, validation of discharge diagnoses, and data analysis.

MC assisted with the study design, served as gold standard panel member, and revised the draft manuscript

VN assisted with study design, served as gold standard panel member, and helped with data analysis

RB assisted with the study design, served as gold standard panel member, and revised the draft manuscript

AW assisted with the study design, served as gold standard panel member, and revised the draft manuscript

HF assisted with data collection and analysis

PT assisted with study conception, study design and revised the draft manuscript

JW assisted with study conception, provided advice regarding study design, and revised the draft manuscript

JB assisted with study conception, study design and data analysis.

All authors read and approved the final manuscript.

## Pre-publication history

The pre-publication history for this paper can be accessed here:


